# Emerging Role of the Macrophage Migration Inhibitory Factor Family of Cytokines in Neuroblastoma. Pathogenic Effectors and Novel Therapeutic Targets?

**DOI:** 10.3390/molecules25051194

**Published:** 2020-03-06

**Authors:** Eugenio Cavalli, Rosella Ciurleo, Maria Cristina Petralia, Paolo Fagone, Rita Bella, Katia Mangano, Ferdinando Nicoletti, Placido Bramanti, Maria Sofia Basile

**Affiliations:** 1 Department of Biomedical and Biotechnological Sciences, University of Catania, Via S. Sofia 89, 95123 Catania, Italy; eugeniocavalli9@hotmail.it (E.C.); paolofagone@yahoo.it (P.F.); kmangano@unict.it (K.M.); sofiabasile@hotmail.it (M.S.B.); 2 IRCCS Centro Neurolesi “Bonino-Pulejo”, Via Provinciale Palermo, Contrada Casazza, 98124 Messina, Italy; rossella.ciurleo@irccsme.it (R.C.); placido.bramanti@irccsme.it (P.B.); 3 Department of Educational Sciences, University of Catania, Via Teatro Greco 84, 95124 Catania, Italy; m.cristinapetralia@gmail.com; 4 Department of Medical and Surgical Sciences and Advanced Technologies, University of Catania, Via S. Sofia 78, 95123 Catania, Italy; rbella@unict.it

**Keywords:** d-dopachrome tautomerase, macrophage migration inhibitory factor, macrophage migration inhibitory factor inhibitors, neuroblastoma

## Abstract

Neuroblastoma (NB) is the most frequent extracranial pediatric tumor. Despite the current available multiple therapeutic options, the prognosis for high-risk NB patients remains unsatisfactory and makes the disease a clear unmet medical need. Thus, more tailored therapeutic approaches are warranted to improve both the quality of life and the survival of the patients. Macrophage migration inhibitory factor (MIF) is a pleiotropic cytokine that plays a key role in several diseases, including cancer. Preclinical and clinical studies in NB patients convergently indicate that MIF exerts pro-tumorigenic properties in NB. MIF is upregulated in NB tumor tissues and cell lines and it contributes to NB aggressiveness and immune-escape. To date, there are only a few data about the role of the second member of the MIF family, the MIF homolog d-dopachrome tautomerase (DDT), in NB. Here, we review the preclinical and clinical studies on the role of the MIF family of cytokines in NB and suggest that MIF and possibly DDT inhibitors may be promising novel prognostic and therapeutic targets in NB management.

## 1. Neuroblastoma (NB)

NB represents the most frequent extracranial pediatric tumor, with an incidence of 10.5 cases per million children among 0 and 14 years of age, in North America and Europe [1]. There are no relevant geographic differences in incidence [1]. However, African American and Native American patients are more likely to have worse outcomes, thus showing ethnic disparities [1]. The majority of NB patients are diagnosed before 5 years of age, with a median age at diagnosis of 19 months [2]. In addition, NB is responsible for 12%–15% of cancer-related mortality in children [1].

NB arises from primordial neural precursor cells of the sympathetic nervous system and mainly develops in the adrenal medulla and/or in the paraspinal sympathetic ganglia of the neck, chest, abdomen or pelvis [3]. About half of patients develop distant metastases and the most frequent metastatic sites are bones, bone marrow, and liver [2].

NB is characterized by heterogeneous clinical course and presentation [4]. While some NBs regress spontaneously, others may aggressively spread [4]. Moreover, signs and symptoms are very different, depending on tumor site and biology and on the eventual presence of metastasis or paraneoplastic syndromes [4].

According to the International NB Staging Series (INSS), which relies on surgical observations, NB is classified by risk level, tumor location and dissemination, and *MYCN* (proto-oncogene protein) amplification [5]. The International NB Risk Group (INRG) Staging System was more recently designed in order to find homogeneous pretreatment risk groups, considering clinical criteria and tumor imaging [6]. The INRG classification takes into account several factors, such as tumor stage and differentiation, patient age, histology, MYCN oncogene status, DNA ploidy, and segmental chromosomal anomalies, in particular chromosome 11q aberration [6]. According to the INRG classification, the patients are stratified in groups with different outcomes and risks including very low, low, intermediate, and high risk [6]. While very low-risk patients have a 5-year event-free survival (EFS) higher than 85%, high-risk patients show a 5-year EFS less than 50% [6].

According to the risk classification, there are different therapeutic approaches for NB patients, such as observation, surgical tumor removal, chemo- and radiotherapy, autologous hematopoietic stem cell transplantation (AHSCT), differentiation therapy, and immunotherapy [7]. In particular, the antidisialoganglioside (anti-GD2) immunotherapy has recently been successfully incorporated into the standard of care treatment for high-risk NB patients [8]. Moreover, a recent randomized clinical trial (NCT00567567) has demonstrated that tandem autologous stem cell transplant with thiotepa/cyclophosphamide followed by carboplatin/etoposide/melphalan resulted in a significantly better EFS than single transplantation with carboplatin/etoposide/melphalan in high-risk NB patients under 30 years of age [9]. Several innovative strategies aimed at targeting the tumor microenvironment, the noradrenaline transporter, and the genetic pathways are being developed with promising effects in NB diagnosis and treatment [7].

Despite these multiple therapeutic options and novel strategies, the prognosis for high-risk NB patients is still unsatisfactory and makes the disease a clear unmet medical need. Therefore, more tailored therapeutic approaches are warranted in order to improve patient survival and quality of life.

## 2. The Macrophage Migration Inhibitory Factor (MIF) Family of Cytokines

### 2.1. MIF

Macrophage Migration Inhibitory Factor (MIF) is a multipotent cytokine discovered in 1966 and is characterized as a T cell-derived mediator, with the peculiar property to inhibit the random movement of macrophages [10].

However, MIF is also expressed by different cell lines such as epithelial, endothelial, and immune cells [11]. Unlike many other cytokines that are secreted upon antigenic stimulation, MIF is constantly expressed and stored in intracellular pools [11]. In addition to cytokine function, MIF also exhibits pleiotropic characteristics of enzyme, hormone, and chaperone protein [11]. MIF plays an important role in the regulation of different physiological functions. Harper et al. reported that MIF regulates energy metabolism through its neuroendocrine effects on insulin signaling pathways in the pancreas, muscle, and adipocytes [12]. Furthermore, MIF has been observed to have effects on the hypothalamic–pituitary–adrenal (HPA) axis. In vivo studies in rodents indicate that MIF is released in association with adrenocorticotropin (ACTH) from the pituitary gland during a period of physiological stress [13]. It was reported that MIF-knockout (KO) mice are fertile, their progeny develop and age normally without showing spontaneous diseases [11]. Moreover, Toso et al. reported in a model that MIF knockout (KO) mice or mice treated with anti-MIF show normal blood glucose levels, lactate response, and liver glycogen content after the administration of endotoxin or TNF-α [14].

MIF activates the signaling complex by binding the protein cluster of differentiation (CD) 74 and the signal transducer CD44 or by interacting with the intracellular receptor JAB1 [15]. At the same time, MIF can activate the family of CXC chemokine receptors (CXCR2, CXCR4, and CXCR7) [15]. The role of the interaction between MIF and CXCR7 through Akt-dependent signaling has been recently studied [16]. MIF receptors establish four different receptor complexes to transduce the signaling pathway: CD74/CD44, CD74/CXCR2, CD74/CXCR4, and CD74/CXCR4/CXCR7 [17]. Genetic ablation or anti-CD74 treatment abolishes MIF signaling in CD44-, CXCR2-, CXCR4-, or CXCR7-expressing cells [17]. CD44 is a co-receptor of CD74 and is pivotal for MIF signal transduction [18]. Upon engagement of MIF with the CD74–CD44 complex, the Src kinase is activated [17], leading to the phosphorylation of the extracellular-signal-regulated kinase ½ (ERK1/2) and the inhibition of tumor suppressor protein 53 (p53) expression [19]. The phosphorylation of extracellular signal-regulated kinase-1 (ERK1) and extracellular signal-regulated kinase-2 (ERK2) of mitogen-activated protein (MAP) kinases is closely related to the transduction of the MIF signal and its interaction with the CD74/CD44 complex [19]. The interaction of MIF and CD74 also promotes the activation of the AKT pathway through the mediation of kinases SRC and PI3K [19,20,21].

The activation of AKT leads to the phosphorylation and inactivation of the pro-apoptotic proteins BCL2 associated agonist of cell death (BAD) and Bcl-2-associated X protein (BAX), allowing the cells to resist apoptosis [20]. Furthermore, in lymphoid cells, the activation of AKT, related to an increase of nuclear factor kappa-light-chain-enhancer of activated B cells (NF-kB) function, promotes the expression of the anti-apoptotic proteins Bcl-xL and Bcl-2 [20].

### 2.2. d-Dopachrome Tautomerase (DDT)

The second member of the MIF family, named DDT or MIF-2, was described in 1997 [22]. Located on the human chromosome 22q11.23, DDT has a homology of 34% with MIF and has a common homotrimer structure [23]. Both homologs have common biological characteristics, such as the enzymatic activity represented by a catalytic proline residue [15]. Like MIF, DDT is able to interact with the CD74 receptor. However, the absence of a binding domain does not allow interaction with CXCR2.

Similarly to MIF, DDT activates the cascade of the MAP kinase ERK1/2 via the activation of the CD74/CD44 complex. This interaction leads to the activation of protein kinase A (PKA), which subsequently phosphorylates SCR and mediates ERK1/2 [17].

It is worth mentioning that DDT was also shown to bind JAB1/CSN5 intracellularly [17].

## 3. The Role of MIF Family in Cancer

### 3.1. MIF and Cancer

We and others have shown that MIF and DDT are involved in several diseases of different origins such as immunoinflammatory and autoimmune diseases, neurodegenerative and neuropsychiatric diseases, and cancer [24,25,26,27,28,29,30,31].

In addition, evidence generated during the last 15 years has also supported a pro-oncogenic role of MIF in certain types of cancers [29,30,31]. MIF may upregulate several tumorigenic processes, including tumor growth, invasiveness, and angiogenesis [32] primarily, but not exclusively, through its angiogenetic action, its AKT mediated antiapoptotic effects, and the inhibition of p53 function.

**Figure 1:**
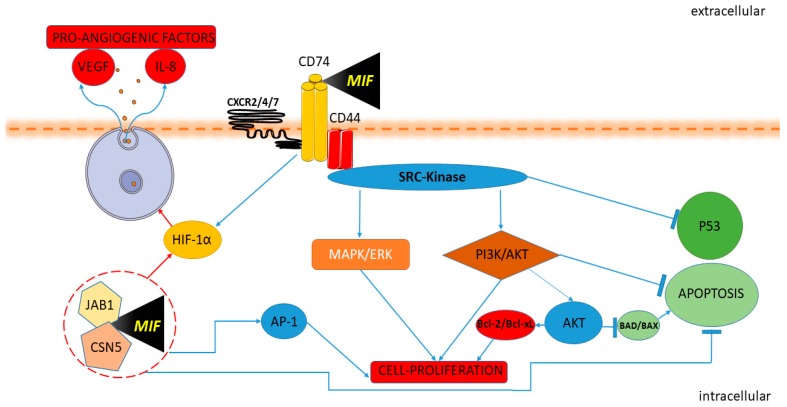
Macrophage migration inhibitory factor (MIF) and cancer pathway. MIF activates the signaling complex binding CD74, CD44 and the chemokine receptors CXCR2, CXCR4, and CXCR7. The Protein Tyrosine Kinase Src is activated by MIF. Upon the engagement of the CD74-CD44 complex, the Src kinase is activated, leading to phosphorylation of the extracellular-signal-regulated kinase ERK/MAP and inhibition of p53 expression. The interaction of MIF and CD74 also promotes the activation of the AKT/PI3K pathway with the consequent inactivation of the pro-apoptotic proteins BAD and BAX and promotes the expression of the anti-apoptotic proteins Bcl-xL and Bcl-2. MIF induces HIF1α activation through extracellular and intracellular interaction. MIF binding to CD74 leads to HIF1α activation. In the intracellular domain, MIF binds Jab1/CSN5, activating HIF1α and leading to the expression of proangiogenic factors IL-8 and VEGF. MIF modulates AP-1 activity and cell proliferation with Jab1/CSN5 interaction and inactivating p53.

Strictly related to vessel growth and cell proliferation is the ability of MIF to activate hypoxia-induced factor-1α (HIF-1α) [33] through the extracellular and intracellular environment [33]. When MIF binds to CD74, it leads to the direct HIF1α activation, while in the intracellular domain, MIF binds Jab1/CSN5, which regulates the functionality of HIF1α by counteracting its hydroxylation and leads to the expression of pro-angiogenic factors, such as IL-8 and vascular endothelial growth factor (VEGF) [33]. At the intracellular level, MIF is also able to modulate AP-1 activity and the cell cycle, synergistically with Jab1/CSN5 interaction, inactivating the tumor suppressor p53 [34] (Figure 1).

Though endowed with proinflammatory activities, several lines of evidence also suggest that at the tumor site MIF may act as a soluble immune checkpoint inhibitor, thus favoring the establishment of immune-evasion in the microenvironment [15] via induction of myeloid-derived suppressor cells [35] and inhibition of T cells activation [36], M1 polarization [37] and reduction of natural killer (NK) cell cytotoxicity [38].

MIF may be implicated in certain forms of tumorigenesis as suggested by genetic studies showing that the single nucleotide polymorphism (SNP) -173 G/C (rs755622) on MIF gene is associated to MIF hyperproduction and correlates with cancer [39].

A meta-analysis by Vera et al. has shown an association between the -173C MIF promoter polymorphism and an increased risk of cancer, particularly for prostate cancer and other solid tumors [40]. Moreover, the genetic polymorphism MIF-173 was associated with a higher risk of early cervical cancer and lymph node metastasis [41] and also with the risk of gastrointestinal cancer and hematological malignancy [42]. In addition, Lin et al. found that MIF rs755622 polymorphism correlated with breast cancer susceptibility in Chinese population, particularly in elderly patients [43].

Numerous preclinical and clinical studies have demonstrated that MIF is overexpressed and may correlate with tumor aggressiveness in many different types of human cancers such as prostate , bladder, and kidney cancer [30], cervical cancer [20], ovarian cancer [44,45], breast cancer [29], gastric cancer [29], hepatocellular carcinoma [46], colon cancer [47,48], pancreatic cancer [49,50], gallbladder cancer [51], lung cancer [29,52], melanoma [31], head and neck cancer [29], acute myeloid leukemia [53,54], glioblastoma [15,55,56], and NB [57,58,59,60]. Moreover, elevated MIF expression is correlated with a worse patient overall survival in a large variety of cancers such as breast cancer [29], gastric cancer [29], hepatocellular carcinoma [61], pancreatic cancer [49,50], metastatic melanoma [31], head and neck cancer [29], esophageal squamous cell carcinoma [62], acute myeloid leukemia [53], glioblastoma [29,56], and NB [57]. However, conflicting results have also been reported and other studies have shown that, in other types of tumors, endogenous MIF may possess a beneficial anticancer activity.

For example, low nuclear MIF expression conferred a poor prognosis to patients with lung adenocarcinoma [52]. Differently, high levels of MIF were correlated with reduced drug responsiveness and with poorer outcomes in lung cancer patients [29]. Conflicting results on the role of MIF in breast cancers have been reported with a study claiming to a correlation between positive MIF expression and a better overall and recurrence-free survival [63] and others demonstrating that positive MIF expression levels correlated with a worse prognosis [64,65]. Contradictory results also exist for colon cancer patients [47] with one study showing that MIF expression was associated with tumor grade and hepatic metastases [66] and another indicating that the elevated MIF expression correlated with better survival in Dukes C or D colorectal tumors [67].

It has also been shown that increased MIF expression in tumor-infiltrating lymphocytes (TILs) within tumor microenvironments was associated with better outcomes in nasopharyngeal carcinoma (NPC) patients [68].

Taken as a whole, these data seem to indicate that the MIF possesses both pro and antioncogenic activities that may depend on the phenotype and site of the tumor and possibly the genetic background of the patients and other yet unidentified cofactors. The data seem to indicate that, for certain types of tumors, both local expression of MIF and its circulating blood levels seem to be promising prognostic and predictive biomarkers and tailored anti-MIF therapies may be beneficial [15].

### 3.2. DDT (MIF2) and Cancer

There are only a few studies on the role of DDT in cancer. It has been shown that the knockdown of DDT and MIF in the pancreatic cell line, PANC-1, correlated with reduced activation of ERK1/2 and AKT, augmented p53 expression, and inhibited tumor growth in vitro and in vivo [15,69]. The DDT interaction with CD74 stimulates the expression of VEGF and CXCL8 and counteracts the 5’ AMP-activated protein kinase (AMPK) activation in human non-small cell lung carcinoma [70,71]. Interestingly, in contrast to the ability of MIF and DDT to activate AMPK in non-transformed cells, they cooperatively inhibited the activation of AMPK in LKB1 mutant human non-small cell lung cancer (NSCLC) cell lines [71]. Furthermore, treatment with the dual inhibitor of MIF and DDT, 4-iodo-6-phenylpyrimidine (4-IPP), decreased in vitro proliferation and in vivo tumor growth in a mouse xenograft model [69]. Moreover, in the melanoma cancer cell line B16F10, treatment with small interfering RNAs (siRNA)/DDT suppressed cell proliferation and stimulated apoptosis, and in a xenograft model treatment with anti-DDT antibodies reduced tumor growth [72]. DDT has also been reported in colorectal cancer, showing to regulate the transcriptional factor β-catenin, in a manner partly dependent on COX-2 expression [73]. In DDT-deficient colorectal cells, the β-catenin expression is reduced [73]. These data suggest that DDT may play an overlapping role with MIF, thus suggesting possible therapeutic actions aimed at inhibiting the two homologs.

## 4. The Emerging Class of Single and Dual Inhibitors of MIF and DDT, from Small Molecules to Biologics, through Drug Repurposing

The data discussed above with the clear-cut efficacy of specific anti-MIF strategies to reduce aggressivity and invasiveness of cancer cells in vitro and in vivo has attracted attention for the adoption of specific MIF and DDT inhibitors for the treatment of cancers [74,75]. Single and dual inhibitors of MIF and DDT are emerging that deserve particular attention in this setting [74,75]. Among MIF- and DDT-targeted pharmacologic approaches there are small molecule inhibitors, monoclonal antibodies, nanobodies, and peptide inhibitors [76]. These compounds have been reviewed extensively and recently elsewhere [74,75,76] and are presented in Table 1.

### 4.1. Small Molecule Inhibitors of MIF and/or DDT

Different categories of MIF inhibitors have been characterized (Table 1). These include the dopachrome analog MIF inhibitors NAPQI, OXIM-11, and DEBIO-1036; two oxazoline derivatives CPSI-2705 and CPSI-1306; the isoxazoline compounds ISO-1, ISO-66, and ISO-92 [77]; and biaryltriazole, pyrazole, and benzoxazol-2-thione [77]. MIF inhibitors identified with in silico methods are the compound Z-590 that inhibits several MIF functions, benzoxazol-2-thione, and ORITA-13; potent inhibitors of the tautomerasic action are 2-oxo-4-phenyl-3-butanoate, phenylpyrimidines, acetaminophen analogs, epicatechins, and the 4-iodo-6-phenylpyrimidine (4-IPP), mainly known for its ability to target both MIF and DDT [15]; a selection of approved drug (ibudilast, ebselen, and iguratimod) [77]; natural products (benzyl isothiocyanate, L/D-thyroxine, ellagic acid, epoxyazadiradione, spirohexenolide-A, a sulfonade organic acid, p425, and a novel isocoumarin compound SCD-19) [76,77,78]. 1,2,3-triazole derivatives have been reported as MIF inhibitors [74].

In addition, a small molecule that selectively inhibits DDT (4-CPPC) has recently been characterized [80].

### 4.2. Biological Inhibitors of MIF and/or DDT

Anti-MIF antibodies are imalumab (Bax69), BaxG03, BaxB01, BaxM159, and milatuzumab that blocks CD74 and hence represents a dual inhibitor of MIF and DDT [15]. A new class of emerging inhibitors of CD74 is peptides DRα1-MOG-35–55, RTL1000, C36L1, and synthetic peptides MIF-(40–49) and MIF-(47–56) that bind CXCR2 [76]. Novel anti-MIF nanobodies are NbE-5, NbE-10, NbH9, and NbE10-Nb Alb 8 [79].

### 4.3. Repurposed Drugs as MIF Inhibitors, the Case of Ibudilast

Ibudilast is a nonselective (3, 4, 10, 11) phosphodiesterase inhibitor that is clinically used as a bronchodilator for the treatment of bronchial asthma. However, recent evidence indicates that this drug possesses a pleiotropic immunopharmacological mode of action that entails, among others, inhibition of tautomerasic activity of MIF and blockade of Toll-like receptor 4. This has propelled several studies aimed at repurposing ibudilast for neuroinflammatory conditions. In particular, a recent Phase II study has shown promising effects of ibudilast in patients with multiple sclerosis [81,82,83]. Other drugs that are being repurposed as MIF inhibitors are ebselen and iguratimod [77].

### 4.4. Inhibiting the MIF Family of Cytokines in Cancer

#### 4.4.1. Chemotherapeutic Action of MIF and/or DDT Inhibitors in Preclinical Studies

Many preclinical studies and a few clinical trials have investigated the effects of several of the abovementioned inhibitors of MIF and/or DDT in different types of cancer.

It has been shown that the tautomerase inhibitor ISO-1 reduced the viability of different cancer cell lines, e.g., prostate adenocarcinoma, lung cancer, and gliomas and has positive effects in vivo in models of melanoma, prostate, and colon cancer [15,74,77]. Furthermore, the ISO-1 derivative ISO-66 reduced tumor growth in murine models of melanoma and colon cancer [15,74]. The oxazoline derivatives MIF inhibitors CPSI-2705 and CPSI-1306 reduced tumor growth and spreading in mouse models of bladder and skin cancer [30,78,84]. Another effective MIF inhibitor was the isocoumarin compound SCD-19, which reduced tumor growth in a murine model of lung cancer [15,74,76]. 4-IPP attenuated tumor growth in head and neck squamous carcinoma and lung adenocarcinoma cells and in in vitro and in vivo models of pancreatic carcinoma [15]. Furthermore, the anti-MIF monoclonal antibodies were effective in vitro and in vivo in prostate cancer and colon cancer models [15,78]. In addition, add on treatment to temozolomide with the MIF inhibitor ibudilast significantly increased the survival in vivo, in a patient-derived xenograft model of glioblastoma [56].

#### 4.4.2. Clinical Studies

Of particular relevance for the purpose of this review is the peculiar highlighting of specific inhibitors of the MIF family of cytokines that have already advanced to the clinical settings or that are already approved for other indications and are being repurposed such as milatuzumab and ibudilast.

A summary of the current clinical trials investigating the role of MIF and MIF inhibitors in cancer is provided in Table 2.

Early phase clinical trials are evaluating anti-MIF therapies for cancer treatment. In particular, the anti-MIF mAb Bax69 was studied in malignant solid tumors (NCT01765790), ovarian cancer (NCT02540356), and metastatic colorectal cancer (NCT02448810) and the anti-CD74 and hence dual inhibitor of MIF and DDT milatuzumab in hematologic malignancies (NCT01101594, NCT00421525, NCT00603668, NCT00868478, NCT00504972, and NCT00989586). Overall, Bax69 and milatuzumab resulted to be well-tolerated. However, their clinical efficacy needs to be ascertained. Currently, two clinical trials aimed to study, respectively, the cotreatment with ibudilast and temozolomide in recurrent glioblastoma patients (NCT03782415), and the anti-CD74 antibody–drug conjugate STRO-001 in patients with advanced B-Cell malignancies (NCT03424603) are recruiting.

## 5. The Role of the MIF Family of Cytokines in NB

The growing body of evidence suggesting a major role for the MIF and DDT in cancer development has attracted interest in the study of its involvement in the pathogenesis and progression of NB. In the remaining part of this review, we will discuss the currently available preclinical and clinical studies on MIF in NB and the unique clinical study on its homolog DDT in NB. Furthermore, we will consider the opportunity to introduce novel MIF- and or DDT-targeting strategies for NB management.

### 5.1. In Silico Analysis of MIF and DDT in NB as Potential Theranostics

DNA microarray analysis is a widely used technique that helps to identify diagnostic tools, pathogenetic pathways, and novel cellular and pharmacological therapeutic targets in several types of pathologies including immunoinflammatory and autoimmune diseases [85,86,87,88,89,90], neurodegenerative diseases [91], and cancer [92,93,94,95] and allows identification of potential cellular and molecular therapeutic targets [96,97].

Along this line of research, we have recently performed an in silico study by interrogating publicly available whole-genome transcriptomic databases in order to evaluate the prognostic property of MIF and of its homolog DDT in NB and to predict a potential therapeutic strategy [57]. We analyzed stage 4 NB samples and we found that patients with higher MIF and DDT expression levels are correlated with a poorer prognosis, independently from MYCN amplification [57]. Furthermore, samples with higher expression of MIF had increased proportions of Th1 cells, while samples with lower MIF expression were enriched in B cells, CD8+ T cells, dendritic, and NK T cells [57]. Overall, our results suggested that MIF and DDT could serve as negative prognostic factors for stage 4 NB patients [57], likely by inhibiting antigen presentation and cytotoxic immune responses [57].

### 5.2. Preclinical Studies

#### 5.2.1. In Vitro Studies

The possible role of MIF in the pathogenesis of NB was first reported by Bin et al. who observed that the murine NB cell line, Neuro2a, secretes MIF. In a similar manner, patient-derived NB cell lines also produced MIF. MIF production by NB was studied at the level of RNA, secreted product by ELISA, and in a macrophage migration assay. NB culture-derived MIF was also shown to activate tumor cell migration. This study first provides in vitro evidence that MIF production is upregulated in NB cell lines and also in NB cells from human patients and put forward the hypothesis that MIF promotes in NB aggressiveness and evasion of immune recognition [60].

A study conducted by Ren et al. reported that MIF was overexpressed in the cytoplasm of NB cell lines (SK-N-SH and SK-N-DZ) and could promote the expression of N-myc [58]. Exposure of NB cell line to MIF induced a significant increase in the activity of map kinases [58]. Pretreatment of the NB cells with the MAP kinase inhibitors PD98059 before MIF stimulation downregulated N-Myc expression in a dose-dependent manner [58].

Fan et al. studied the impact of MIF on the expression of genes in the NB SK-N-AS cell line [98] and reported a change in expression of several genes with a significant upregulation of 99 genes and a downregulation of 24 genes [98]. Among the oncogenes, growth factors, and pro-metastatic genes, such as cell division cycle 34, kruepel-subfamily C2H2-type zinc finger protein upregulated [98]. It was also reported that the upregulation of type IV collagen and inter-α-trypsin inhibitor heavy chain was associated with tumor metastasis [98].

Liu et al. have shown that the transfection of the SK-N-SH and GI-LA-N human NB cell lines with miR-451 reduced tumor proliferation, invasion, and migration, and that MIF was negatively regulated by miR-451 that directly targeted the 3′UTR of MIF mRNA in the NB cell lines [99]. Moreover, the overexpression of MIF counteracted the inhibitory action of miR-451 on NB cells growth, invasiveness, and migration, thus indicating that the anticancer activity of miR-451 may be due to the suppression of MIF expression [99].

Since the histone deacetylase (HDAC) inhibitor vorinostat that inhibits MIF [100] induces cell death in NB cell lines [101], we have recently performed an in silico analysis in order to investigate the potential involvement of MIF and DDT modulation in the in vitro anticancer activity of this drug in NB patients [57]. Interestingly, we found that the expression levels of MIF and DDT in the NB cell line, SH-SY5Y, were significantly reduced after treatment with vorinostat, thus suggesting that HDAC inhibitors may be a possible novel therapeutic approach for NB patients [57].

#### 5.2.2. In Vivo Studies

The oncogenic potential of MIF that stemmed from the above-mentioned in vitro observations has been subsequently strengthened by in vivo studies that convergently provided clear-cut evidence for the pathogenic effect of MIF in NB development.

To evaluate whether the reduction of MIF expression could inhibit cell proliferation and tumorigenicity of NB [102], the NB cells (SK-N-DZ) were transfected with antisense (AS) MIF. The cells with diminished MIF production exhibited lower expression of the c-Met, N-Myc, TrkB, Ras, and IL-8 along with upregulated expression of tumor repressor genes, i.e., EPHB6 and BLU [102]. In addition, when nude mice were inoculated subcutaneously with either empty vector-transfected NB cells (control group) or AS-MIF-transfected NB cells groups, the former group developed lung metastasis at a higher percentage and more rapid kinetic than those of the latter group [102].

#### 5.2.3. MIF as a Novel Immune Checkpoint Inhibitor in NB?

Although MIF has primarily been regarded as a proinflammatory cytokine, the study of its role in oncogenesis has revealed an unexpected role of this cytokine as a promoter of immune suppression. The first in vivo evidence in this regard stems from the observation that anti-MIF treatment in mice xenografted with the NB cell lines, EG7, showed increased T cell accumulation in the tumor [103].

This observation generated interest in the possibility that MIF may represent an additional immune checkpoint inhibitor that may favor development, maintenance, and progression of NB. The area of research of specifically enforcing immune responses in NB as novel therapeutic approaches has recently gained much attention in light of recent promising effects observed with different immunotherapeutic approaches in NB patients.

An anti-GD2 vaccine in association with beta-glucan is in phase II clinical trial, and the effects of nivolumab and ipilimumab are being evaluated in recurrent tumors. To aid prediction of clinical responses to immunotherapy, we recently generated a computational model integrating the different intracellular pathways involved in NB in order to predict the sensitivity to anti-programmed cell death-ligand-1 (PD-L1) immunotherapy [104]. Yan et al. have first shown that upregulated production of MIF from the murine NB cell line Neuro-2a suppressed T-cell activation. Furthermore, the inhibitory effects of culture supernatants from NB were reversed when the cells were transfected with MIF si-RNA. It was proposed that overproduction of MIF from NB cells provokes activation-induced T-cell death through an IFN-gamma pathway that may eliminate activated T cells from the tumor microenvironment and thus contribute to escape from immune surveillance [36].

The hypothesis that MIF might represent an important immune checkpoint inhibitor in the pathogenesis of NB was further strengthened by subsequent studies in murine models of NB. It was reported that the MIF knockdown in AGN2a NB cells elicited a stronger T cell-dependent rejection than that of control cells. Tumors originating from MIF KO AGN2A cells showed increased T cell infiltration, along with higher numbers of macrophages, dendritic cells, and B cells. Immunization with MIF KO AGN2a cells significantly increased protection against tumor as compared with immunization with wild-type AGN2a cells, increasing the proportions of tumor-specific cytotoxic T cells. However, the finding that addition of anti-MIF Abs to the AGN2a culture supernatants was not sufficient to inhibit the T cell suppressive effects suggests that MIF may not be sufficient in vivo for immune suppression. This suggests that in NB MIF expression secondarily activates an immunosuppressive pathway that leads to the inhibition of T cell immunity [59].

Another study conducted by Fan et al. investigated if and how influencing MIF expression was implicated in the development of NB [105]. The expression of MIF was reduced by transfection of the antisense MIF pSec vector into NB cell line SK-N-DZ [105]. By using microarray technology it was reported that in MIF-reduced NB cells there was a downregulation of certain genes associated with tumor development including IL-8 and C-met along with upregulation of the tumor-suppressor genes EPHB6, visinin-like protein 1 (VSNL-1), and BLU [105]. Consistently with these observations, when SN-K-DZ with reduced MIF expression were transplanted into nude mice their growth was significantly lower than that observed in the cells of control animals that were transplanted with SN-K-DZ cells transfected with the vectors alone [105].

#### 5.2.4. Clinical Studies

Intracellular MIF was found to be overexpressed in tumor tissues of NB patients and significantly correlated with the grade of tumor differentiation and N-Myc expression [58]. Ren et al. analyzed the association of MIF and c-Met in tumor specimens from NB patients by immunohistochemical staining and they found that the expression of MIF was significantly positively correlated with the expression of c-Met, thus suggesting that MIF could be involved in the upregulation of c-Met expression [102].

Liu et al. have shown that MIF was a direct target gene of miR-451, which was significantly downregulated in tumor tissue samples of NB patients [99]. Moreover, they found that the reduction in miR-451 was significantly associated with negative tumor features, such as higher TNM stage, larger carcinoma size, lower degree of differentiation, and higher presence of metastases [99]. Therefore, they suggested that the miR-451/MIF pathway could be a novel therapeutic target for NB patients [99].

## 6. Single and Dual Inhibitors of MIF and DDT for the Treatment of NB. What Is at the Horizon?

Along with the converging in vitro and in vivo evidences highlighting a multifaceted pathogenetic role of MIF and DDT in NB, attention is warranted on the possibility to use the abovementioned single and dual inhibitors of MIF and DDT in NB patients and, with particular high urgency, in those cases that are poor responders to SOC treatment. Although it has been shown that several MIF inhibitors, such as the oxazoline derivatives, have good safety profiles in vivo, they may exert potential immunosuppressive side effects [78,106]. Considering that there are different functional *MIF* promoter variants that could be considered as a genetically defined therapeutic window, the anti-MIF therapy might improve reducing MIF expression to the level present in low-genotypic MIF expressers [106]. Moreover, since MIF may exert a pro- or anti-oncogenic action depending on the type of tumor and on the microenvironment, in order to overcome potential side effects, it could be useful to consider the membrane permeability of any MIF inhibitors and to take into account its role in intracellular and extracellular spaces [78]. Of major relevance for the immediate translation to the clinical setting are those specific MIF and DDT inhibitors listed in Table 1 that are already in the clinical phase of development or, and even more, that have already been approved for different indications. These include the single MIF inhibitor Bax69 that is in Phase I/II a studies in different forms of cancer, the dual MIF and DDT inhibitor milatuzumab that is approved for hematological malignancies [107], and ibudilast.

## 7. Conclusions

Although there are multiple therapeutic approaches and novel advances, NB still represents a huge unmet medical need. Further studies are needed in order to clarify the biological mechanisms involved in NB pathophysiology and in order to identify novel tailored therapeutic strategies, thus improving patient survival and quality of life. A growing body of evidence suggests that MIF and probably DDT could be involved in tumorigenesis by inhibiting antigen presentation and cytotoxic immune responses and favoring tumor evasion from immune recognition.

**Figure 2:**
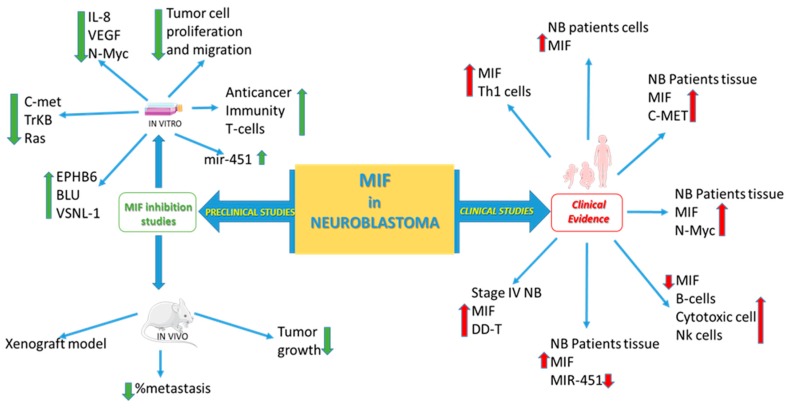
MIF in neuroblastoma (NB).

This review highlights the involvement of the MIF family of cytokines in the development of NB. Preclinical in vitro and in vivo studies and clinical studies in NB patients convergently indicate that the MIF family of cytokines exerts pro-tumorigenic properties in NB (Figure 2).

Preclinical and clinical studies on MIF in NB show the results obtained in in vitro and in vivo studies and the correlation between MIF overexpression and NB in patients.

The current studies agree that MIF is upregulated in NB tumor tissues and cell lines and that it may be involved in NB aggressiveness and immune-escape. To date, there are only a few lines of evidence about the role of the second member of the MIF family of cytokine, DDT, in NB. In particular, it has been demonstrated that, along with MIF, DDT could be a negative prognostic factor for stage 4 NB patients.

The involvement of MIF and of its homolog DDT in NB tumorigenesis has a potentially significant impact for this area of research since it might open novel promising therapeutic and diagnostic possibilities, including the opportunity to administer MIF and/or DDT inhibitors as an eventual complementary therapy along with the NB standard of care treatment.

In addition to the abovementioned specific MIF family inhibitors, another possible valuable opportunity could be to use semi-specific MIF inhibitors, such as HDAC inhibitors. Indeed, it has been recently found that the expression levels of MIF and DDT were significantly reduced in NB cell lines after treatment with the HDAC inhibitor vorinostat [57].

Moreover, it has been found that nitrosylation could dampen MIF activity [108]. Hence, another possible interesting approach may be to use nitric oxide (NO)-hybridized drugs, such as NO-aspirin or NO-hybridized antiretroviral protease inhibitors, including lopinavir-NO [87,92,94,95] and ritonavir-NO [87,93], for the treatment of NB and eventually other diseases in which MIF is involved.

Overall, we suggest that the MIF family of cytokines is involved in the pathogenesis of NB and that MIF and DDT could be promising theragnostic cytokines that may be useful in NB management either as prognostic or therapeutic targets.

## Figures and Tables

**Table 1:** MIF family inhibitors.

**SPECIFIC** **INHIBITORS**	**Single** **Activity**	**Small molecules** [15,74,75,76,77,78]		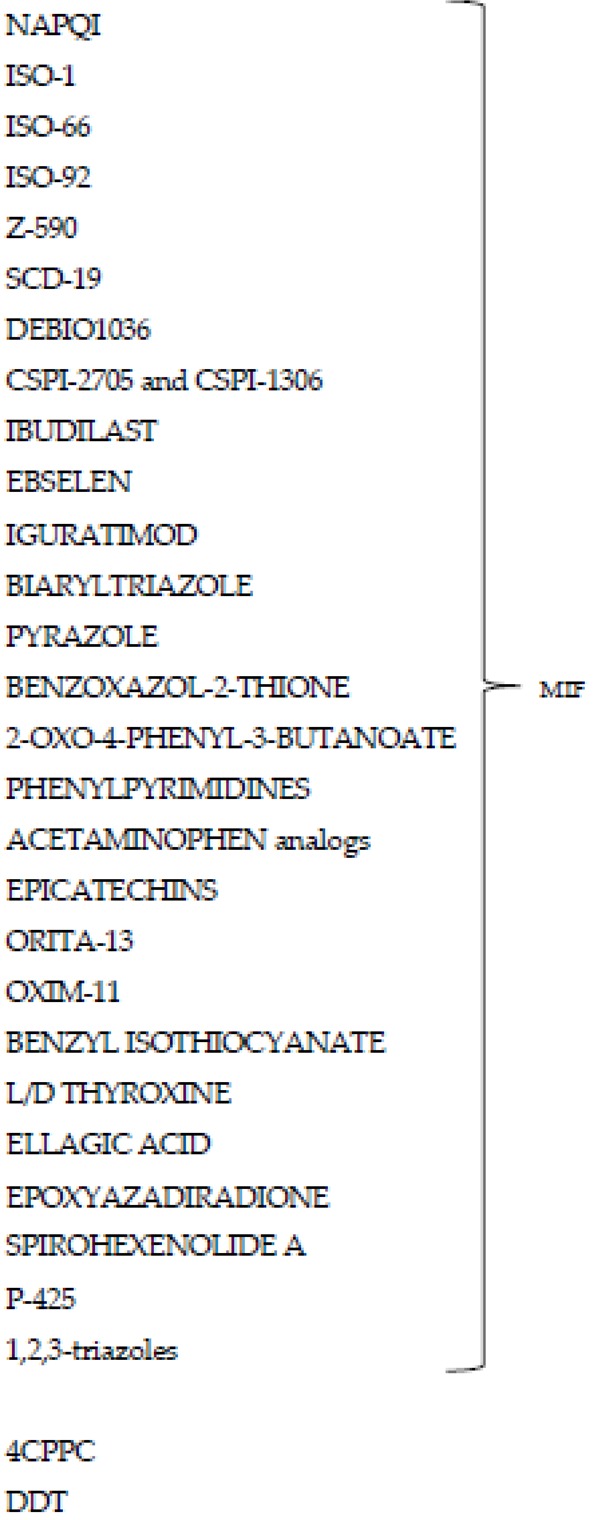
		**Biologics**	**Peptides** [76]	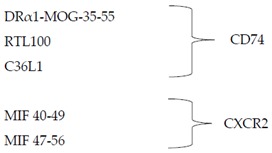
			**Monoclonal antibodies** [15,76,78]	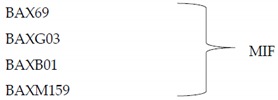
			**Nanobodies** [79]	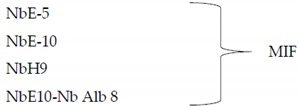
	**Dual activity**	**Small molecules** [76]		4-IPP MIF/DDT
		Antibodies [76]		MILATUZUMAB CD74/MIF/DDT

**Table 2:** MIF and d-dopachrome tautomerase (DDT) inhibitors in clinical trials.

**Anti-MIF Interventions**				
**Title**	**Status**	**Conditions**	**Interventions**	**Identifier**
Phase 1 Study of Anti-Macrophage Migration Inhibitory Factor (Anti-MIF) Antibody in Solid Tumors	Completed	- Metastatic Adenocarcinoma of the Colon or Rectum - Malignant Solid Tumors	- Biological: Anti-Macrophage Migration Inhibitory Factor (Anti-MIF) Antibody	NCT01765790
Phase 1/2a Two-Arm Dose-Escalation Study of BAX69 in Subjects With Malignant Ascites of Ovarian Cancer	Terminated	- Refractory Ovarian Cancer With Recurrent Symptomatic Malignant Ascites	- Biological: BAX69 Single-Route Arm - Biological: BAX69 Double-Route Arm	NCT02540356
Phase 2a Study of BAX69 and 5-FU/Leucovorin or Panitumumab Versus Standard of Care in Subjects With Metastatic Colorectal Cancer	Terminated	Metastatic Colorectal Cancer	- Biological: BAX69 + infusional 5-FU/LV - Biological: BAX69 + panitumumab - Biological: BAX69 + 5-FU/LV - Drug: Standard of Care - Biological: Standard of Care	NCT02448810
Study to Evaluate Ibudilast and TMZ Combo Treatment in Recurrent GBM	Recruiting	- Glioblastoma - Recurrent Glioblastoma - GBM - Recurrent GBM	- Drug: MN-166 - Drug: Temozolomide	NCT03782415
**Anti-CD74 Interventions**				
**Title**	**Status**	**Conditions**	**Interventions**	**Identifier**
Study of STRO-001, an Anti-CD74 Antibody Drug Conjugate, in Patients With Advanced B Cell Malignancies	Recruiting	- B-cell Lymphoma - Non-Hodgkin Lymphoma - Multiple Myeloma - Follicular Lymphoma - Mantle Cell Lymphoma - Diffuse Large B Cell Lymphoma - Indolent Lymphoma - B Cell Tumors	- Drug: STRO-001	NCT03424603
Phase I Trial of Anti-CD74 (hLL1) Antibody Therapy in B Cell Malignancies	Completed	- Non-Hodgkin’s Lymphoma - Chronic Lymphocytic Leukemia	- Drug: milatuzumab	NCT00504972
A Study of hLL1-DOX (Milatuzumab-Doxorubicin Antibody-Drug Conjugate) in Patients With Multiple Myeloma	Completed	Multiple Myeloma	- Drug: hLL1-DOX (the doxorubicin conjugate of milatuzumab)	NCT01101594
Phase I/II Study of hLL1 in Multiple Myeloma	Completed	- Multiple Myeloma - Myeloma, Plasma Cell -PLASMACYTOMA	- Biological: milatuzumab	NCT00421525
Veltuzumab and Milatuzumab in Treating Patients With Relapsed or Refractory B Cell Non-Hodgkin Lymphoma	Completed	Lymphoma	- Biological: milatuzumab - Biological: veltuzumab - Procedure: Correlative/Special Studies - Procedure: Quantitative T-, B-, and NK cell subsets - Procedure: Pharmacokinetics - Procedure: Human Anti-Human Antibodies - Biological: veltuzumab and milatuzumab	NCT00989586
Phase I/II Study of Different Doses and Dose Schedules of Milatuzumab (hLL1) in CLL	Completed	Chronic Lymphocytic Lymphoma	Biological: milatuzumab	NCT00603668
The Humanized Monoclonal Antibody Milatuzumab for Refractory Chronic Lymphocytic Leukemia (CLL)	Unknown status	Chronic Lymphocytic Leukemia	Drug: Milatuzumab	NCT00868478
